# Segmental ureterectomy does not compromise the oncologic outcome compared with nephroureterectomy for pure ureter cancer

**DOI:** 10.1007/s11255-013-0514-z

**Published:** 2013-11-08

**Authors:** Shih Ya Hung, Wen Chou Yang, Hao Lun Luo, Chun-Chien Hsu, Yen Ta Chen, Yao Chi Chuang

**Affiliations:** Department of Urology, Kaohsiung Chang Gung Memorial Hospital, 123, Ta-Pei Road, Niaosung, Kaohsiung, Taiwan

**Keywords:** Ureter, Urothelial carcinoma, Segmental ureterectomy, Nephroureterectomy

## Abstract

**Purpose:**

Pure ureter cancers are rare and account for only 1–3 % of urothelial carcinomas with limited data. Nowadays, nephron-sparing methods are reserved mainly for imperative cases. This study intends to assess the oncologic outcome between segmental ureterectomy (SU) and radical nephroureterectomy (RNU) for pure ureteral urothelial carcinoma.

**Methods:**

From July 2004 to August 2010, 112 patients at a single tertiary referral center were included. Perioperative data were obtained from our institutional database. Postoperative CT scan, cystoscopy, and contralateral renal echo were performed regularly for survey of disease recurrence.

**Results:**

The mean length of follow-up was 43.8 and 48.3 months for the RNU and SU group, respectively. The bladder recurrences, local recurrences, distant metastasis, and cancer-specific survival rates showed no significant differences between RNU and SU (36.4 vs. 34.2 %, *p* = 0.83; 23.4 vs. 14.3 %, *p* = 0.27; and 16.9 vs. 8.6 %, *p* = 0.244, and 13.0 vs. 5.7 %, *p* = 0.249, respectively).

**Conclusion:**

The study suggested that SU is not inferior to RNU for ureter cancer in oncologic outcomes and is less invasive and better nephron preservation.

## Introduction

Upper urinary tract urothelial carcinomas (UUT-UC), especially pure ureter cancers, are rare and account for only 1–3 % of urothelial carcinomas [1], and limited data about pure ureter cancers have been reported. Although the concept for preservation of renal function has been emphasized nowadays, radical nephroureterectomy with excision of the bladder cuff (RNU) remains the gold standard treatment for upper urinary tract urothelial carcinomas (UUT-UCs) [2]. Nephron-sparing methods such as endoscopic ablation or segmental ureterectomy (SU) are reserved for imperative cases such as renal insufficiency, solitary functional kidney, low-grade, and low-stage tumors [3–5]. Recent studies supporting oncologic outcomes of SU were comparable to RNU in select cases [4, 6, 8]. This study intends to assess the oncologic outcome between SU and RNU in patients with ureter urothelial cancer treated at a single tertiary referral center.

## Materials and methods

From July 2004 to August 2010, 112 patients with pure ureter cancer were included in this study. All patients received CT scan and cystoscopy for diagnosis of distant metastasis or concurrent bladder tumor; 77 patients underwent RNU and 35 patients underwent SU (Table 1). Segmental ureterectomy was performed under good protection of surrounding tissue to prevent local seeding of urothelial tumors. Radical nephroureterectomy was performed along with standard en bloc bladder cuff excision. Perioperative data were obtained from our institutional database. SU was indicated in cases as patients with chronic renal insufficiency, solitary kidney, and patients’ request. There were no statistically significant differences between age, gender, smoking, tumor grade, preoperative eGFR, advanced pathology (stage more than T2 or positive lymphovascular invasion), and bladder cancer histories. Postoperative CT scan, cystoscopy, and contralateral renal echo were performed regularly for survey of disease recurrence. None of these patients received adjuvant chemotherapy or radiotherapy. The postoperative eGFR measured at 1 year after surgery. When comparing the perioperative eGFR change, we excluded patients with end-stage renal disease. Software SPSS version 17 was used for statistical analysis in this study. Chi-square and two-sample *t* test were used for intergroup comparison, while Kaplan–Meier survival plot was used for comparison of recurrence-free survival or cancer-specific survival. Statistical significance was set if *p* value was <0.05.

**Table 1:** Patient characteristics

	RNU	SU	*p* value
Patient number	77	35	
Follow-up duration (months)	43.84 ± 20.64	48.26 ± 26.97	0.344
Age	66.71 ± 9.96	69.29 ± 9.44	0.201
Male/female	41/36	18/17	0.858
High grade	68 (88.3 %)	30 (85.7 %)	0.700
Multifocality	17 (22 %)	2 (5.7 %)	0.032
Preoperative eGFR	54.60 ± 28.78	56.31 ± 33.62	0.522
Postoperative eGFR change	−10.66 ± 24.5	1.18 ± 14.9	0.011
Bladder cancer history	16 (20.8 %)	8 (22.9 %)	0.804
Non-organ confined (>T2)	15 (19.5 %)	11 (31.4 %)	0.165
Bladder recurrence	28 (36.4 %)	12 (34.2 %)	0.832
Local recurrence	18 (23.4 %)	5 (14.3 %)	0.270
Distant metastasis	13 (16.9 %)	3 (8.6 %)	0.244
Cancer death	10 (13.0 %)	2 (5.7 %)	0.249

## Results

**Fig. 1:**
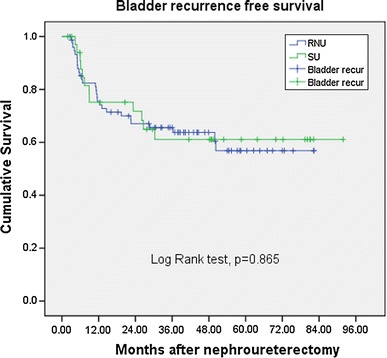
Bladder recurrence free survival

**Fig. 2:**
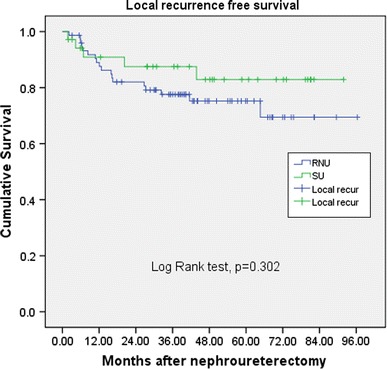
Local recurrence free survival

**Fig. 3:**
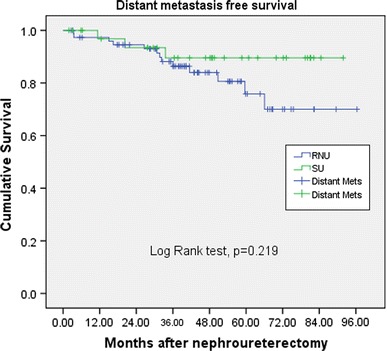
Distance metastasis free survival

**Fig. 4:**
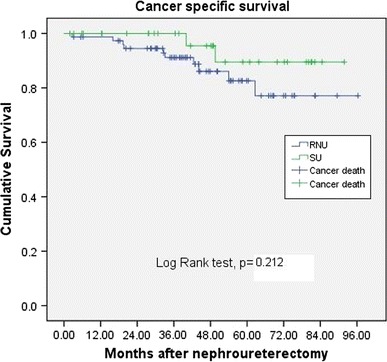
Cancer specific survival

The patients were mainly elderly in this study with median age being 68 years old. The mean length of follow-up was 43.8 and 48.3 months for the RNU and SU group, respectively (*p* = 0.344) (Table 1). There were 17 patients in the RNU group had multifocality, and 2 patients in the SU group had multifocal lesions (*p* = 0.032). Both groups has similar ratio of patient with bladder cancer history, 21 % in RNU group and 23 % in SU group (*p* = 0.804). The bladder recurrences rates, local recurrences rates, distant metastasis rates in univariate analysis showed no significant differences between RNU and SU (36.4 vs. 34.2 %, *p* = 0.83; 23.4 vs. 14.3 %, *p* = 0.27; and 16.9 vs. 8.6 %, *p* = 0.244, respectively). No significant differences existed between surgical intervention with regard to cumulative bladder recurrence-free survival (Fig. 1, *p* = 0.865); local recurrence-free survival (Fig. 2, *p* = 0.302); distant metastasis-free survival (Fig. 3, *p* = 0.219), and cancer-specific survival (Fig. 4, *p* = 0.212). The postoperative eGFR improved 1.19 ± 14.94 ml/min/1.73 m^2^ in SU group, and the postoperative eGFR decreased 10.66 ± 24.49 ml/min/1.73 m^2^ in RNU group. The RNU group had worse eGFR after operation than SU group (*p* = 0.011). There were higher local recurrence rate in multifocal lesions patients (*p* = 0.047, HR 3.9, 95 % CI 1.1–14.8). When divided the patients into advanced stage (>T2) and non-advanced stage (≦T2) in multivariate analysis, there were higher local recurrences (*p* < 0.001. HR 9.4, 95 % CI 2.8–31.8), distant metastasis (*p* = 0.002, HR 11.2, 95 % CI 2.5–50.8), and cancer-specific survival rates (*p* = 0.07, HR 8.8, 95 % CI 1.8–43.2) in advanced stage patients. Patients with bladder cancer histories had higher bladder recurrence rate (*p* = 0.025, HR 3.2, 95 % CI 1.2–8.6). Otherwise, smoking patients had more bladder recurrence (*p* = 0.033, HR 3.7, 95 % CI 1.1–11.9) and distance metastasis (*p* = 0.012, HR 6.2, 95 % CI 1.5–25.6) (Tables 2, 3).

**Table 2:** Multivariate analysis for bladder and local recurrence

	Bladder recurrence		Local recurrence	
	Univariate *p* value	Multivariate *p* value	Univariate *p* value	Multivariate *p* value
Surgical intervention RNU/SU	0.832		0.270	
Organ confinement >T2/≤T2	0.894		<0.001	<0.001 HR 9.4, 95 % CI 2.8–31.8
Tumor grade High/low	0.551		0.185	
Smoking Yes/no	0.035	0.033 HR 3.7, 95 % CI 1.1–11.9	0.187	
Multifocality Yes/no	0.027	0.074	0.001	0.047 HR 3.9, 95 % CI 1.1–14.8
Bladder cancer history Yes/no	0.009	0.025 HR 3.2, 95 % CI 1.2–8.6	0.968

**Table 3:** Multivariate analysis for distant metastasis and cancer death

	Distant metastasis		Cancer death	
	Univariate *p* value	Multivariate *p* value	Univariate *p* value	Multivariate *p* value
Surgical intervention RNU/SU	0.244		0.249	
Organ confinement >T2/≦T2	0.006	0.002 HR 11.2, 95 % CI 2.5–50.8	0.002	0.007 HR 8.8, 95 % CI 1.8–43.2
Tumor grade High/low	0.414		0.166	
Smoking Yes/no	0.023	0.012 HR 6.2, 95 % CI 1.5–25.6	0.212	
Multifocality Yes/no	0.837		0.016	0.474
Bladder cancer history Yes/no	0.301		0.287	

## Discussion

Upper urinary tract cancer, especially pure ureter cancer, is a rare malignancy. RNU with bladder cuff removal remains the gold standard treatment for ureter cancer [2]. However, according to the reviewed literature, patients with UUT-UC having high prevalence of chronic kidney disease and renal function often deteriorated after RNU [9, 10]. Ou et al. [11] hypothesized that the uremic environment may be a significant factor promoting the underlying urinary tract disease to develop cancers. To preserve renal function, nephron-sparing methods such as endoscopic ureter tumor excision and SU were developed, which are alternative choices of treatment for imperative indication [5, 12]. More and more series have indicated the important role of nephron-sparing methods for ureter cancer patients even with contralateral normal kidney or locally advanced pathological features [4, 9, 13]. Further validation about the safety of SU is worthy of investigation.

Nephron-sparing surgeries for ureter cancer such as endoscopic ureter tumor excision and SU have been reported to have acceptable oncologic outcome. Endoscopic ureter tumor excision has even been reported to manage early stage and superficial tumor, but the under-staging and increased risk of recurrences were noted [14]. However, even for muscle invasive ureter cancer, SU could possibly achieve en bloc resection of the ureter tumor with surrounding tissue. Preoperative diagnosis of muscle invasive ureter cancer is the key point to decide whether endoscopic or segmental ureterectomy should be safely performed. Several series have attempted to develop nomograms based on preoperative imaging, tumor grade from ureteroscopic biopsy, architecture, and location for the prediction of non-organ-confined UUT-UC [15, 16]. It is still difficult to differentiate between muscle invasive and non-muscle invasive diseases preoperatively. SU seems to be more suited to ureter tumor excision without the risk of ureter perforation by endoscopic excision and thus has routinely been performed in our institution for patients willing to preserve the kidneys. Considering the bias from the open or endoscopic approach and different surgical techniques by surgeons, this study only assessed the oncologic outcome of SU compared with RNU by a single tertiary center experience.

NCCN guidelines for upper urinary tract cancer suggest standard nephroureterectomy with bladder cuff excision for high-grade mid-ureter cancer and all upper ureter cancer [7]. Distal ureterectomy with ureter reimplantation could be considered for clinically feasible patients. However, Colin et al. reported a multicenter cohort study of ureter cancer which suggested that tumor location and surgical interventions were not an independent prognostic factor for recurrence-free survival or cancer-specific mortality [8]. In addition, the 5-year probability of CSS, RFS, and MFS for SU and RNU were 87.9 and 86.3 % (*p* = 0.99); 37 and 47.9 % (*p* = 0.48); and 81.9 and 85.4 % (*p* = 0.51), respectively. Jeldres et al. also reported the oncologic outcome among SU, RUN with bladder cuff removal, and RNU without bladder cuff removal. The 5-year cancer-specific mortality-free rates were 86.6 versus 82.2 versus 80.5 %, respectively, without significant differences. The populations of these studies were relatively unequal in patient numbers because of the retrospective design. These results are similar to our observations. Our result revealed that advanced pathology of ureter cancer is common, occupying 19.5 and 31.4 % in RNU and SU groups, respectively. However, cancer-specific survival was similar between SU and RNU in both non-advanced and advanced pathology of pure ureter cancer patients in our study. Kaplan–Meier plot also revealed that SU did not increase the disease recurrences compared with RNU.

Although in the study, we noted that pathological stage is still the most important predictor for local and distant oncologic failure with subsequent cancer-specific death. The evidence we reviewed support that SU is not inferior to RNU even for locally advanced ureter cancer, which is at high risk for adjuvant chemotherapy. Despite adequate radical surgery, disease recurrence is not uncommon for UUT-UC. For distant metastasis or unrespectable disease, cisplatin-based chemotherapy is now the current treatment of choice. The advantage of SU is kidney preservation with better renal function outcome and then increased eligibility for adjuvant chemotherapy [12]. In our study, not only the RNU group had significant eGFR decrease, but also the SU group showed improvement in renal function. Jonathan L. et al also mentioned about this phenomenon may contribute to the resection of a ureteral tumor with obstructive uropathy [17]. In addition, CKD is well known to cause more cardiovascular disease and subsequent mortality [18]. The actual benefit of SU upon CKD prevention for patients with ureter cancer and its correlation between cancer-specific or overall survival benefit is worthy of further investigation.

Through multivariate analysis, we reveled that urinary bladder cancer historied are independently associated with urinary bladder tumor recurrence. And also smoking is a related factor to urinary bladder recurrence and distant recurrence. Maurice et al. [19] reported approximately threefold higher risk of urinary tract cancer in cigarette smokers than non-smokers.

The limitations of this study are its retrospective, non-randomized design, and relatively small sample size. There were unequal case numbers for multifocal tumor, and it is reasonable because that multifocal tumor is not always suitable for nephron-sparing method. Thus, we used multivariate analysis to identify the impact of cancer control. Most of our multifocal cases received SU because of chronic renal insufficiency or multiple comorbidity so that patients decided to have nephron-sparing surgery and our data supported that there was no significant difference in bladder recurrence, distant recurrence, or cancer-specific survival.

However, the advantage is single tertiary center experiences without bias from patient population and basic surgical procedures; especially in Southern Taiwan, we have a higher prevalence of upper urinary tract cancers in black foot endemic areas [20]. We present the result with detailed pathology review and patient follow-up. The intention is to help validate the role of SU in treating patients with ureter urothelial cancer.

## Conclusion

The study suggested that SU is not inferior to RNU for ureter cancer in oncologic outcome. In addition, SU is less invasive and has better nephron preservation compared with RNU. Further randomized studies with larger cohort series are still necessary to support the results.

## Abbreviations

UUT-UC: Upper urinary tract urothelial carcinomas
RNU: Radical nephroureterectomy
SU: Segmental ureterectomy
